# An irregular atrial tachycardia: What is the underlying mechanism?

**DOI:** 10.1007/s12471-017-1050-8

**Published:** 2017-10-27

**Authors:** S. Pagano, G. Aguglia, D. Noto, M. Averna

**Affiliations:** 1 Sezione di Medicina Interna e Malattie Metaboliche, Dipartimento di Medicina Interna e Specialistica, DIBIMIS, Università di Palermo, Palermo, Italy; 2 Dirigente Medico presso MCAU, ARNAS Civico, Palermo, Italy

## Answer

**Fig. 1:**
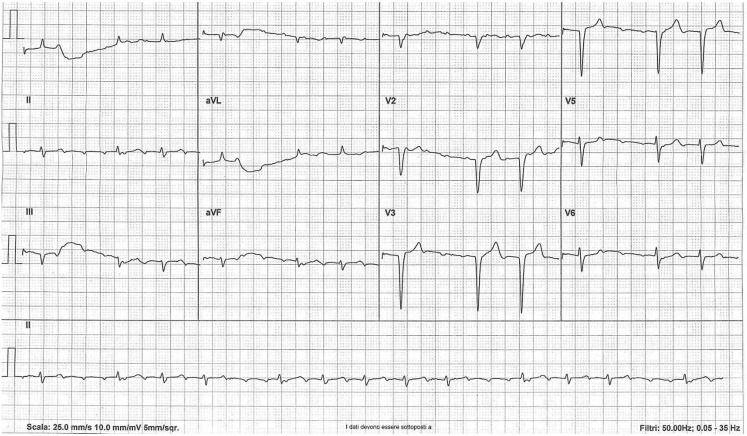
The irregular atrial tachycardia

There are clearly visible *P*-waves in most of the leads (Fig. 1). The *P*-waves are negative in leads DII, DIII and aVF. Hence, this is not a sinus rhythm. All the *P*-waves have the same morphology, constant PP interval at a rate of 210 beats per minute (bpm), with an isoelectric baseline between them. Therefore, the underlying rhythm is unifocal atrial tachycardia [1].

**Fig. 2:**
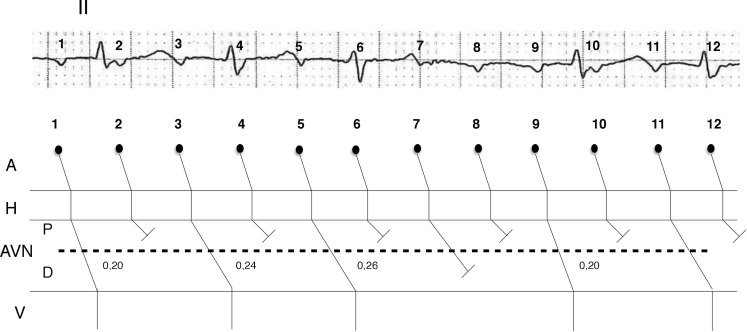
The alternating Wenckebach phenomenon visible on the DII lead (see text for discussion) (*A* atria, *AVN* atrioventricular node, *P* proximal block, *D* distal block, *V* ventricles, *1–12* *P*-waves)

Lead DII shows 12 *P*-waves, but some of these waves are barely discernible because they are superimposed on the QRS-T complexes (Fig. 2). In the first part of the strip there are *P*-waves alternately conducted and non-conducted to the ventricles, characterising a 2:1 AV conduction. In the middle half of the strip there is a longer RR interval where only the ninth *P*-wave conducts to the ventricles, while the seventh, eighth and tenth *P*-waves are blocked, realising a 4:1 AV conduction. So, the atrial tachycardia presents an alternating 2:1 and 4:1 AV conduction. The long RR interval is less than twice the short RR intervals and the PR intervals progressively lengthen until a pause with 3 non-conducted *P*-waves occurs. These elements are consistent with alternating Wenckebach periodicity, a rare phenomenon characterised by a block in two levels of the atrioventricular node; one proximally, giving rise to a 2:1 block and one distally, responsible for the Wenckebach periodicity that explains the progressive PR lengthening until the non-conducted *P*-wave [2]. Alternating Wenckebach periodicity is encountered primarily in atrial tachyarrhythmias; the most frequent block level is the atrioventricular node, but it has been described in almost every level of the conduction pathways, including accessory ones [3].
